# The Impact of Perioperative Remote Patient Monitoring on Clinical Staff Workflows: Scoping Review

**DOI:** 10.2196/37204

**Published:** 2022-06-06

**Authors:** Maria Alejandra León, Valeria Pannunzio, Maaike Kleinsmann

**Affiliations:** 1 Department of Design, Organization and Strategy Faculty of Industrial Design Engineering Delft University of Technology Delft Netherlands

**Keywords:** remote patient monitoring, telemonitoring, workflow, nurses, physicians, perioperative care, perioperative medicine, telehealth, mobile phone

## Abstract

**Background:**

Remote patient monitoring (RPM) interventions are being increasingly implemented in health care environments, given their benefits for different stakeholders. However, the effects of these interventions on the workflow of clinical staff are not always considered in RPM research and practice.

**Objective:**

This review explored how contemporary RPM interventions affect clinical staff and their workflows in perioperative settings.

**Methods:**

We conducted a scoping review of recent articles reporting the impact of RPM interventions implemented in perioperative settings on clinical staff and their workflow. The databases accessed were Embase and PubMed. A qualitative analysis was performed to identify the main problems and advantages that RPM brings to staff, in addition to the approaches taken to evaluate the impact of those interventions. Different themes were identified in terms of the challenges of RPM for clinical staff as well as in terms of benefits, risk-reduction strategies, and methods for measuring the impact of these interventions on the workflow of clinical staff.

**Results:**

A total of 1063 papers were found during the initial search, of which 21 (1.98%) met the inclusion criteria. Of the 21 included papers, 15 (71%) focused on evaluating new RPM systems, 4 (19%) focused on existing systems, and 2 (10%) were reviews.

**Conclusions:**

The reviewed literature shows that the impact on staff work experience is a crucial factor to consider when developing and implementing RPM interventions in perioperative settings. However, we noticed both underdevelopment and lack of standardization in the methods for assessing the impact of these interventions on clinical staff and their workflow. On the basis of the reviewed literature, we recommend the development of more robust methods for evaluating the impact of RPM interventions on staff experience in perioperative care; the adoption of a stronger focus on transition management when introducing these interventions in clinical practice; and the inclusion of longer periods of assessment, including the evaluation of long-term goals.

## Introduction

### Background

Remote patient monitoring (RPM) interventions allow patients to be continuously monitored at a distance and beyond the physical borders of the hospital or health care institution [1]. RPM interventions have been used to monitor patients within clinical settings (eg, in intensive care environments) or outside of care facilities (eg, in the patients’ homes). Moreover, RPM has been used for delivering care for multiple health conditions, from heart failure [2] to diabetes [3] and skin problems [4].

RPM interventions can provide 24-hour care as they can collect data continuously and alert specialists when certain parameters are outside the standard thresholds [5]. This can enable real-time adjustments, timely decisions, and improved care. RPM as a field has also enjoyed an unprecedented acceleration as a consequence of the COVID-19 pandemic, which has stimulated the adoption of remote care to minimize face-to-face interactions between patients and staff [6]. In the perioperative setting, RPM can be useful for assessing physical conditions preoperatively or monitoring patients’ recovery after discharge. Although RPM applications in this domain are still relatively novel, encouraging results are driving an increased interest from researchers and practitioners.

An example of the application of RPM technologies to perioperative care was offered by Atilgan et al [7], who evaluated a system comprising monitoring devices collecting several vital signs (including blood pressure, heart rate, oxygen saturation, body temperature, blood glucose, and electrocardiography) and a mobile app providing medication reminders, suggested daily life activities, diet and nutrition plans, and web-based visit capabilities. Vital parameters were measured in patients who had undergone cardiac surgery after discharge and automatically transferred to a telemedicine team for assessment. Overall, the authors reported the RPM intervention to have resulted in high patient satisfaction, prevention of incorrect medications and dosages, prevention of rehospitalization, and early detection of potentially life-threatening complications.

Much of the available research on RPM interventions in the perioperative domain focuses on the effects of RPM on patients [8-11] and describes its advantages, especially in terms of clinical outcomes and efficiency gains [12-14]. Some studies have also addressed the benefits for health care providers, such as hospitals, nursing homes, and other entities. These studies tend to focus on the economic benefits for providers, for instance through reductions in hospitalizations and thus, in the use of resources [15,16]. However, there is limited knowledge of the benefits and limitations of RPM for clinical staff.

### Objectives

This research seeks to evaluate the impact of RPM interventions on the workflow of clinical staff in the context of perioperative care. To explain what we mean by *workflow*, we follow Carayon et al [17], who defined workflow as “the flow of people, equipment, information, and tasks, in different places, at different levels, at different timescales continuously and discontinuously, that are used or required to support the goals of the work domain.” This means that we aimed to evaluate the impact of RPM-related tasks in combination with previously existing activities. In this paper, the words *clinical staff* will be used when referring to both nurses and specialists. To investigate the impact of RPM on the workflow of clinical staff, a human factor perspective was adopted in this review. As mentioned by Hignett et al [18], human factors help in understanding the interactions between humans and the elements of a system to optimize its performance and human well-being.

This scoping review sought to answer the following overall research question: What is the impact of perioperative RPM interventions on the workflow of clinical staff? To answer this main question, we developed the following subresearch questions: (1) What are the problems and challenges of perioperative RPM interventions for clinical staff from a workflow perspective? (2) What are the benefits of perioperative RPM interventions for clinical staff from a workflow perspective? (3) What strategies are implemented or proposed to overcome the problems that perioperative RPM interventions present to the workflow of clinical staff? (4) How is the impact of perioperative RPM interventions on the workflow of clinical staff evaluated and measured?

## Methods

### Overview

This scoping review followed the PRISMA-ScR (Preferred Reporting Items for Systematic Reviews and Meta-Analyses extension for Scoping Reviews) checklist [19]. As the review focuses on collecting and comparing workflow-related insights from recent RPM literature rather than on drawing conclusions on specific outcomes, the risk of bias in the results of the included studies was not assessed. Conversely, the risk of bias in the synthesis of the literature review findings was considered. Specifically, the risk of bias owing to missing results was assessed by MAL and VP through the framework for assessing the risk of bias owing to missing results in a synthesis offered in the Cochrane Handbook for Systematic Reviews of Interventions [20]. The results of this assessment are discussed in the *Limitations* section.

### Selection Criteria and Search Strategy

The databases used were PubMed and Embase. To define the inclusion criteria, key concepts were selected. For each of them, keywords were defined to guide the search strategy (Textbox 1). For the keywords of each concept, the logical operator *OR* was included to consider all the possibilities, whereas the logical operator *AND* was used between concepts. The full queries in both databases are presented in Multimedia Appendix 1. Finally, the search included articles that were written in English between January 2015 and March 2021. This was chosen to obtain a picture of contemporary RPM interventions as this review focuses on current challenges and opportunities. The search was conducted during the last week of March 2021.

The articles resulting from this search were screened based on the following inclusion criteria: (1) the inclusion of RPM interventions for perioperative care and (2) the mention of the impact on the workflow of clinical staff.

The criteria were used for 2 iterations of screening: the first was based on the title and abstract of the articles, and the second was based on the full text.

**Concepts included in the literature search.**

- A keyword can have some variations (plural or singular form or simple or continuous verb form). An asterisk (*) is used for the search algorithm in the database to find all possible variations of a certain word.
  Remote patient monitoring: remote monitor*; telemedicine; telemonitoring; telehealth, remote follow-up; eHealth; remote consultation; remote sensing technology; self-monitor*
  Workflow: workflow; outcome and process assessment, health care; task performance and analysis; workflow; staffing; attitude of health personnel; alarm fatigue*; alert fatigue; professional burnout, workload; patient care management; nursing process*; clinical competence; caregiver burden; time and motion studies; work simplification; practice patterns, nurses; nursing audit
  Perioperative care: surgical procedures, operative; general surgery; perioperative; surgery; post-operative; post-discharge

### Review Process and Analysis

Our main categories were established (Textbox 2) to analyze the studies, namely challenges and problems, benefits, risk-reduction strategies, and evaluation methods. These were based on the main goals of this research and the research questions.

The articles were reviewed by MAL, who was also responsible for data extraction. Subsequently, the first step of the analysis was performed by classifying the results into the chosen categories. The second step consisted of creating different themes per category. This step required several iterations to obtain the final set of themes.

**Categories used for data extraction.**

- Problems and challenges of remote patient monitoring (RPM) interventions for clinical staff: includes the problems shown regarding RPM interventions for clinical staff.
- Benefits of RPM interventions for clinical staff: includes the benefits concerning RPM interventions for clinical staff.
- Risk-reduction strategies regarding RPM interventions for clinical staff: includes solutions tested to tackle some of the problems brought by the introduction of RPM interventions and some of the proposals suggested.
- Methods to measure and quantify the impact of RPM interventions on clinical staff: includes the methods used to determine the impact of RPM interventions on clinical staff’s tasks and workflow. It entails the variables and measures collected and analyzed.

## Results

### Overview

**Figure 1:**
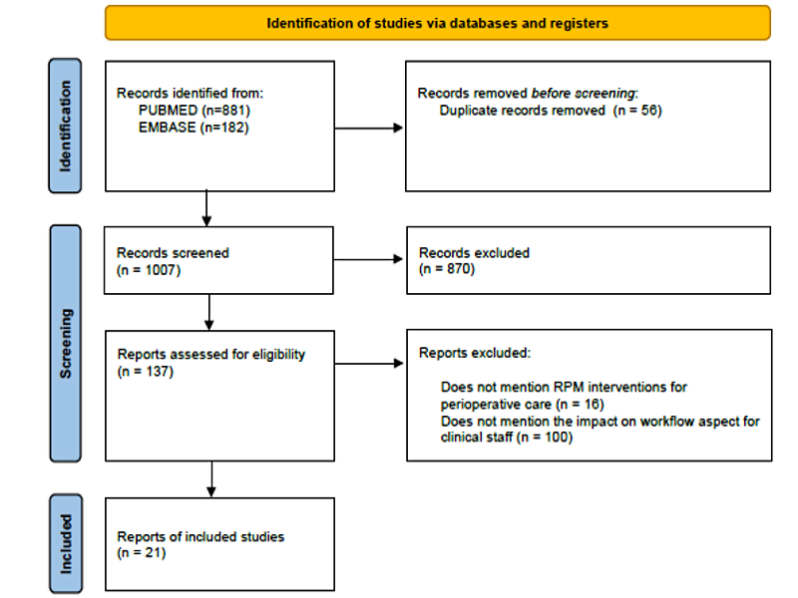
Flowchart of the scoping review process and the inclusion and exclusion criteria. RPM: remote patient monitoring.

A total of 1063 articles were identified after searching both databases, of which 1007 (94.73%) were left after deduplication. Of these 1007 articles, 137 (13.6%) fulfilled the first round of selection, and 21 (2.09%) passed the final round of selection (Figure 1).

In general, the articles included in this review were experimental or observational studies. Of the 21 articles, 15 (71%) involved the evaluation of a design intervention (an RPM model, tool, or service), 4 (19%) consisted of an analysis of already implemented interventions, and the remaining 2 (10%) were reviews. The references and articles analyzed in these 2 reviews did not include any of the other selected articles in this scoping review.

The studies focused on a wide range of patient cohorts and surgical specialties, including orthopedic, bariatric, and oncological surgery. Most of these studies (20/21, 95%) focused on adult patients (aged >18 years). The described RPM interventions ranged from 1 to 45 months of duration.

In addition, the articles presented different types of RPM interventions, ranging from e-tools used only by the clinical staff to services and models that incorporated devices and platforms for both patients and specialists. Moreover, most of the interventions contemplated nursing staff as the main actors responsible for remote care and included physicians for specific tasks or just in case a more detailed and in-depth analysis of the patient’s data was needed.

Not all the included studies contained information on all the categories established. For example, the included reviews hardly mentioned the methodologies used to assess the impact of different RPM interventions on the workflow of the clinical staff.

**Figure 2:**
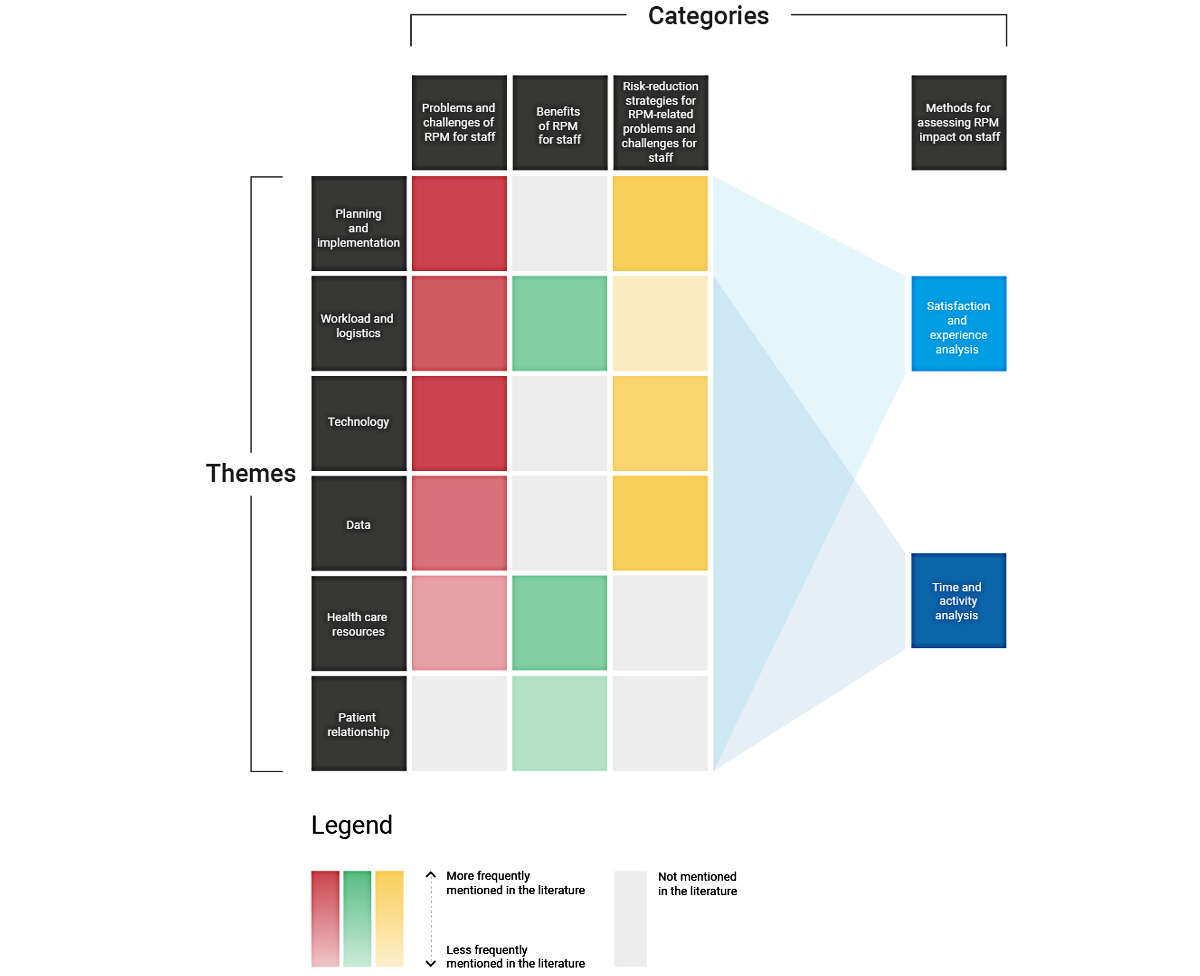
Heat map of the review results organized by categories (each corresponding to a research question) and themes (recurring topics touched on in the included studies). RPM: remote patient monitoring.

Once the data were extracted from the articles, they were classified into the 4 categories. To better understand each category, different themes were defined (Figure 2) based on the similarity of the topics addressed in each of the articles. Figure 2 presents an overview of this classification, where each category is labeled with a different color. By means of a gradient in the color’s intensity, it is possible to show the quantity of papers that touch on each of the proposed themes. In this case, more saturated colors represent more papers mentioning information relevant to a specific theme. The results for each category are discussed in detail in the following sections.

### Category 1: Problems and Challenges of RPM for Clinical Staff

On the basis of the articles analyzed, 5 main themes regarding RPM challenges from the viewpoint of clinical staff were identified (Table 1). The first theme was planning and implementation. Planning is a complex task in health care given the diversity of the stakeholders involved and their needs. RPM projects do not always involve or consider the complex context in which these interventions have to be implemented. This often leads to ambiguity in tasks and roles and, thus, to lack of clarity and structure in the workflow of the clinical staff.

The second theme was workload and logistics. Some staff members do not feel comfortable with the new *behind-the-desk* activities, which can result in unpredictable and emergent tasks when RPM systems register values outside the thresholds. Moreover, data analysis may require more than one specialist, making the workflow more complex. In addition, RPM is perceived as bringing more work, which adds to the existing schedule.

The third theme was technology. Systems might not be user-friendly, and different technical malfunctions may arise, which may require extra expertise from clinical staff.

The fourth theme was data, which can produce more informed decisions but also increase time and be burdensome to analyze. Moreover, it can be hard to keep all the data under 1 platform, so the staff may need to analyze multiple fragments of information to provide remote care.

The last theme was health care resources, intended as the new resources that RPM interventions require. Moreover, the aforementioned ambiguity in tasks determines a lack of clarity regarding reimbursement policies.

A detailed overview of the reported challenges for each category is provided in Table 1.

**Table 1:** Overview of problems and challenges of remote patient monitoring (RPM) interventions for clinical staff.

Theme and description		Studies
**Planning and implementation**		
	Lack of previous user testing	Harsha et al [21]
	Lack of planning or inadequate planning Lack of contemplation of changes in workflow (tasks, competences, responsibilities, and roles) Emergence of uncontemplated tasks No standardization in practices and no clear guidelines Noncompatibility with current practices No clear definition of time for tasks No long-term care coordination Services are implemented before all the resources are available and prepared	Das et al [22] Davoody and Hägglund [23] Harsha et al [21] Ke et al [24] Leppla et al [25] Sanger et al [26] Timmerman et al [27] Wiadji et al [28]
	Lack of resource analysis (“readiness level”) No clear overview of required skills No consideration of staff experience No clarity on resource accessibility (whether clinical staff is adequately equipped)	Ke et al [24] Parkes et al [29] Rothgangel et al [30] Wiadji et al [28]
	Lack of multidisciplinary awareness Uncontemplated users, nonusers, and other actors affected Limited or poor communication and coordination among users Poor task planning (tasks overlapping and no consideration for the need of staff to attend to 1 patient at a time) Disregard for the specificities of different specialties and wards (eg, cardiovascular and pediatric)	Harsha et al [21] Leppla et al [25] Makhni et al [31] Parkes et al [29] Wiadji et al [28]
	Lack of compliance and engagement Lack of involvement of stakeholders in planning Fear of conflict of interest Lack of promotion and motivation among staff Decrease of use of systems over time Resistance to change Specialists and rural hospitals, among others, feeling threatened to be replaced	Downey et al [32] Harsha et al [21] McMullen et al [33] Parkes et al [29] Rothgangel et al [30] Sharif et al [34] Timmerman et al [27] Wiadji et al [28]
**Workload and logistics**		
	High workload New tasks as an addition and not a replacement Telehealth tasks are perceived to be labor-intensive (“More administrative work in arranging telehealth than meets the eyes”) Tracking patients takes too much time (because of subtasks such as setting up appointments, billing, mailing, analyzing, reviewing transmissions, documenting in the EMR^a^, and physician contact) Remote patients are not considered as part of “normal flow” (ignored for workload calculation) Potentially adding an unnecessary step when patient attention is needed (immediate patient check by GP^b^ instead of data follow-up by nurse) Documentation is burdensome	Brophy [35] Das et al [22] Dunphy et al [36] Harsha et al [21] Ke et al [24] Leppla et al [25] Makhni et al [31] McMullen et al [33] Parkes et al [29] Sharif et al [34] Wiadji et al [28]
	Disruption in workflow Unpredictable, emergent tasks High memory load Mistakes on interrupted activities Unanswered or unplanned calls	Das et al [22] Downey et al [32] Harsha et al [21] Sanger et al [26]
	Nonurgent tasks emerge outside working hours	Ke et al [24]
	Need of trustworthy professionals for data analysis Nurses sometimes need to consult with physicians	Leppla et al [25]
	Fear of infringing on other providers’ patient care	Brophy [35]
	Stress because of pressure for timely responses to multiple issues	Das et al [22] McMullen et al [33] Parkes et al [29]
**Technology**		
	Difficulties in use of e-tools Not user-friendly No experience or training	Brophy [35] Das et al [22] Davoody and Hägglund [23] Parkes et al [29] Rothgangel et al [30] Sousa et al [37] Timmerman et al [27]
	Technical problems Troubleshooting and malfunctions Connection issues (eg, congestion, no signal, and delays) Not compatible with current software	Augestad et al [38] Brophy [35] Harsha et al [21] Makhni et al [31] Timmerman et al [27]
	Deficient communication Inappropriate means of communication Hard to establish “personal connection” for communicating bad news or managing conflict with patients New medical-legal situations (patients might misunderstand information or take it out of context) RPM interventions might not be suitable to all the patients	Augestad et al [38] Dunphy et al [36] Ke et al [24] Leppla et al [25] Makhni et al [31] Parkes et al [29] Wiadji et al [28]
	RPM does not offer monitoring to the same extent as in-hospital monitoring No physical examination Cannot assess if patient does self-monitoring or prescribe activities correctly	Dunphy et al [36] Ke et al [24]
**Data**		
	False or insignificant alarms or overreaction Stress by constant sound Turning devices off or not using them	Downey et al [32] Harsha et al [21] Richards et al [39]
	Unclear data and meaning Require extensive analysis Overabundance of data No flag data Missing connection among data	Das et al [22] Leppla et al [25] Sharif et al [34]
	No clear “holistic” impression of patients Lack of data integration with EMR and other existing platforms Not all the reports generated by the system are consulted by physicians	Das et al [22] Semple et al [40] Sharif et al [34] Timmerman et al [27]
	Low reliability of patient monitoring Incomplete data Incorrect measurements	Leppla et al [25] Sharif et al [34]
	Legal issues (eg, privacy, firewall, and licenses)	Brophy [35] Das et al [22] Ke et al [24] Makhni et al [31] Semple et al [40]
**Health care resources**		
	Lack of funding Higher costs than budget Nonsustainable billing rates No clinic income established Higher payment for in-hospital visits	Das et al [22] Brophy [35] Harsha et al [21] Makhni et al [31] Wiadji et al [28]
	Demand of new or more resources	Das et al [22] Makhni et al [31]
	Difficult to quantify quality and effort	Wiadji et al [28]
	Unclear compensation or reimbursement policies Telehealth can take up the same amount of time for significantly less remuneration	Brophy [35] Ke et al [24] Semple et al [40]

^a^EMR: electronic medical record.

^b^GP: general practitioner.

### Category 2: Benefits of RPM for Clinical Staff

For the *benefits* category, 3 main themes were identified as relevant (Table 2). The first theme was the improvement that RPM brings regarding workload and logistics as it allows for the definition of guidelines for more consistent care pathways. This also includes improvements in data management and analysis, which produces timely detection and treatment of conditions.

The second theme was health care resources, which can be operated more effectively with the reduction of in-hospital visits and stays and the possibility of extending coverage of care.

Finally, patient relationship can be improved by increasing satisfaction and convenience of care.

**Table 2:** Overview of benefits of remote patient monitoring interventions for clinical staff.

Theme and description		Studies
**Workload and logistics**		
	Care pathways are standardized More systematic and consistent activities	Brophy [35] McMullen et al [33]
	Reducing the incidence of duplicate documentation	Jansson et al [41]
	Promote collaboration among health care specialists New and appropriate means to hold clinical meetings Patient information can be made accessible to the caregivers involved	Sharif et al [34] Wiadji et al [28]
	Reduce time to reach a clinical decision Shortens face-to-face consultation time Patients are better prepared for the appointment	Ke et al [24] Sharif et al [34]
	Improve sense-making of data Include more sources for analyzing patients’ clinical condition (current state, feedback, and patients’ experience and feeling) Reassuring system based on predefined algorithms for clinical support and suggestions Increased detection of events Real-time monitoring of symptoms over a prolonged period	Jansson et al [41] Ke et al [24] Leppla et al [25] Makhni et al [31] McMullen et al [33] Parkes et al [29] Richards et al [39] Sharif et al [34] Timmerman et al [27]
**Health care resources**		
	Reduce workload	Leppla et al [25] Parkes et al [29]
	Can reduce costs Prevents unnecessary visits and health care use Reduces tests and investigations	Augestad et al [38] Makhni et al [31] Parkes et al [29] Sharif et al [34]
	Increase accessibility More patients can be taken care of More hospitals (eg, rural and remote) can track patients Customizable service (awareness of unique individual challenges)	Augestad et al [38] Brophy [35] Das et al [22] Davoody and Hägglund [23] McMullen et al [33] Timmerman et al [27]
	Allow for a new form of triage for better assessment of patients and resource allocation	Sharif et al [34] Wiadji et al [28]
**Patient relationship**		
	Increase patient satisfaction and convenience	Augestad et al [38] Dunphy et al [36] Parkes et al [29] Sharif et al [34]
	Increase awareness of patient’s daily life	Das et al [22] Davoody and Hägglund [23]

### Category 3: Risk-Reduction Strategies Regarding RPM for Clinical Staff

This category is about strategies to overcome and minimize the challenges that RPM interventions bring about to clinical staff (Table 3). For ease of reference, we refer to risk-reduction strategies related to the introduction of RPM interventions as *strategies*. First, we listed strategies regarding planning and implementation of RPM interventions. Most of the included studies (14/21, 67%) mentioned the value of involving the relevant stakeholders in these processes to understand their needs and the repercussions of the introduction of the RPM intervention on their workflow. Stakeholders’ involvement and participatory approaches were also deemed useful to assess the resources necessary for RPM interventions, the possible risks associated with them, and the need for possible changes to the implementation plans. Finally, training and establishment of protocols (regarding activities, communication, time, and resources) help in risk reduction during implementation and increase the chances of success and adoption.

Second, we listed strategies regarding workload and logistics. Several included studies (8/21, 38%) suggested the creation of new roles for nurses and teams for the remote care of patients, where specialists would be consulted only in special cases. Some strategies to avoid an increase in workload for nursing staff included facilitating collaboration between actors and helping them plan their tasks.

The third theme was technology, which should be user-friendly, interoperable with existing devices and systems, and allow for automatic data collection.

Finally, we identified the theme of data. To avoid the analysis of RPM data being burdensome for staff, smart systems based on customizable alerts were proposed to prevent resource overuse and the incidence of false alarms. These should include measurements from different devices or sources and be presented to the relevant staff in an actionable and understandable way to avoid extra time and burden.

**Table 3:** Overview of risk-reduction strategies regarding remote patient monitoring (RPM) interventions for clinical staff.

Theme and description			Studies
**Planning and implementation**			
	Develop an integrated governance structure Involve all actors concerned with patient management (co-design and participatory practices) Set clear objectives, success metrics, and methods to measure them	Das et al [22] Harsha et al [21] Ke et al [24] Leppla et al [25] McMullen et al [33] Parkes et al [29] Sanger et al [26] Semple et al [40] Timmerman et al [27]	
	Determine health care resource use in terms of the following: Clinical staff and skills Tasks and their timing (to avoid invisible or additional work, time, roles or teams, or an inadequate alert response) Awareness of the multidisciplinary environment Plan for problem solving and changes needed Time for solving technical or general problems Devices and structure	Brophy [35] Das et al [22] Ke et al [24] Leppla et al [25] Parkes et al [29] Richards et al [39] Timmerman et al [27] Wiadji et al [28]	
	Define practice standards, policies, and best practices in terms of the following: Workflow Documentation Communication pathways Measurements Types of data collected Impact on the clinical staff’s well-being (clinical staff’s attitudes, performance, and overall service satisfaction)	Augestad et al [38] Das et al [22] Harsha et al [21] Jansson et al [41] Ke et al [24] Leppla et al [25] Sanger et al [26] Semple et al [40] Timmerman et al [27] Wiadji et al [28]	
	Risk assessment Perform adequate device testing Contemplate technical or general problems (extra time)	Brophy [35] Das et al [22] Leppla et al [25] Richards et al [39] Timmerman et al [27]	
	Consider current state and context Plan according to resources, program, location, dynamics (within the hospital and among clinical staff), and schedules (consider “less busy” and “very busy” times) Customize interventions for integration with existing clinical dynamics and tools	Brophy [35] Das et al [22] Davoody and Hägglund [23] Jansson et al [41] McMullen et al [33] Richards et al [39] Sousa et al [37]	
	Definition of reimbursement policies Automatically track time for standardization Consider financial or nonfinancial options (awards and acknowledgments) Automatically measure time to determine billing Include billing functionalities in the intervention	Das et al [22] Wiadji et al [28]	
	Training staff on tools and protocols Promote enthusiasm, value, and importance among medical staff regarding RPM	Brophy [35] Das et al [22] Downey et al [32] Jansson et al [41] Leppla et al [25] Makhni et al [31] McMullen et al [33] Rothgangel et al [30] Semple et al [40] Sousa et al [37] Timmerman et al [27] Wiadji et al [28]	
**Workload and logistics**			
	Devise a primary nursing-based model (physicians for emergencies and medical decisions)	Leppla et al [25]	
	Allow for easy collaboration between the different actors	Davoody and Hägglund [23] Leppla et al [25]	
	Create dedicated teams for RPM interventions	Leppla et al [25]	
	Include planning tools for routines and tasks Define goals for tasks to make progress clear	Davoody and Hägglund [23]	
	Externalize tasks Have specialized centers for data analysis and alarm reviews	Leppla et al [25]	
	Ensure accessibility to patients’ contact details (to facilitate appointment scheduling and remote consultations)	Jansson et al [41] Ke et al [24]	
	Make e-tools available in different languages	Brophy [35]	
**Technology**			
	Provide appropriate support and access to software and technology for both patients and specialists Ensure compatibility with different smartphones and tablets	Dunphy et al [36] Rothgangel et al [30] Wiadji et al [28]	
	Ensure QoS^a^ support	Harsha et al [21]	
	Integrate with current technologies Interoperable and compatible with other or existing devices and systems Guarantee a seamless connection between RPM platform and staff’s EMR^b^ system	Harsha et al [21] Leppla et al [25] McMullen et al [33] Rothgangel et al [30]	
	Ensure automatic measurements and documentation	Das et al [22] Ke et al [24] Sanger et al [26]	
	Develop user-friendly tools for clinical staff and patients	Augestad et al [38] Brophy [35] Davoody and Hägglund [23] Leppla et al [25] McMullen et al [33] Timmerman et al [27]	
**Data**			
	Alert-based follow-up protocol Continuous data collection (24-hour data) but data analysis focused on alerts by patient prioritization and event-triggered assessment (identify main events to follow) Automatic event classification and suggestions for corrective actions Providing memory aids to staff for interrupted tasks	Dunphy et al [36] Ke et al [24] McMullen et al [33] Richards et al [39] Sanger et al [26]	
	Customizable data collection According to treatment, acuity, goal, progress, and diagnosis (identify high-risk patients to determine extra measures needed)	Das et al [22] Davoody and Hägglund [23] Downey et al [32] Jansson et al [41] Ke et al [24] McMullen et al [33] Rothgangel et al [30]	
	Present easy-to-interpret and actionable data Filter data (“noise cancellation” and false positives) Provide comparison of individual scores with “standard values” of comparable patients	Dunphy et al [36] Leppla et al [25] McMullen et al [33] Rothgangel et al [30] Sanger et al [26]	
	Incorporate different kinds of measurements (from different physiological variables) Include historical patients’ data	Davoody and Hägglund [23] Dunphy et al [36] Jansson et al [41] McMullen et al [33] Rothgangel et al [30] Sanger et al [26]	
	More effective use of patients’ data Use RPM data to guide future medical appointments Use RPM data to assess eligibility for procedures, possible risks, and outcomes	Dunphy et al [36] Jansson et al [41] Parkes et al [29] Sharif et al [34] Wiadji et al [28]	
	Collect data on patient and staff feedback on the intervention for improvement purposes	Jansson et al [41] Leppla et al [25]	
	Provide patients with tools to help assess, interpret, and act upon symptoms	Leppla et al [25]	

^a^QoS: quality of service.

^b^EMR: electronic medical record.

### Category 4: Methods to Measure and Quantify the Impact of RPM on Clinical Staff

This category presents the methods used to identify the impact of RPM interventions on clinical staff tasks and workflows. In total, 2 main themes were established (Table 4) based on the kind of measures of the impact of RPM interventions on staff being collected and analyzed using different methods. The first theme was time and activity analysis, which includes methods for measuring clinical staff time expenditure and workload in relation to existing activities and RPM interventions. These methods allow for a comparative analysis between the standard of care and the RPM intervention. Other possible quantifiable measures found in this category are the number of times certain resources are accessed or the time spent on certain tasks.

The second theme was staff satisfaction and experience, which focuses on how RPM interventions are perceived by the staff and how the new tools and ways of working affect their behaviors. This theme includes subjective measures, such as those gathered through interviews and surveys, and more objective measures, such as measures of adherence to protocols or alert frequency.

**Table 4:** Overview of methods to measure and quantify the impact of remote patient monitoring (RPM) interventions on clinical staff.

Theme and description			Studies
**Time and activity analysis**			
	Activity timing Automatic recording of time spent on events and consultations Duration of use of RPM tools Cumulative time on activities Cumulative time on platform Frequency and quantity of alerts	Downey et al [32] Makhni et al [31] Rothgangel et al [30] Sousa et al [37] Timmerman et al [27] Wiadji et al [28]	
	Activity mapping Current state mapping Implementation assessment Number of times telemonitoring tools were used Number of transmissions and events Selecting most busy times Nurses’ tasks	Augestad et al [38] Leppla et al [25] Rothgangel et al [30] Sousa et al [37] Timmerman et al [27]	
	Comparative analysis with baseline (time spent on activities and number of in-hospital visits and events)	Harsha et al [21] Sousa et al [37]	
	Hospital logistics Number of in-hospital visits Length of in-hospital visits Type of complications Type of resources Accessibility to resources (quantity and quality)	Augestad et al [38] Downey et al [32] Rothgangel et al [30]	
	Cost savings based on time and resources used	Makhni et al [31]	
**Satisfaction and experience analysis**			
	Surveys and questionnaires Usability (eg, System Usability Score) Adherence to protocols Utility and efficiency of e-tools (frequency of incomplete data and effort and work needed for gathering extra data)	Downey et al [32] Leppla et al [25] McMullen et al [33] Parkes et al [29] Rothgangel et al [30] Timmerman et al [27] Wiadji et al [28]	
	Interviews and focus groups	Das et al [22] Davoody and Hägglund [23] Downey et al [32] Dunphy et al [36] Jansson et al [41] Ke et al [24] Leppla et al [25] McMullen et al [33] Parkes et al [29] Sharif et al [34]	
	Ethnographic research Observation Journey mapping	Augestad et al [38] Das et al [22] Leppla et al [25] McMullen et al [33]	
	Co-design and cocreation sessions and workshops Critical incident technique—think-aloud approach—mock-ups	Sanger et al [26] McMullen et al [33] Rothgangel et al [30]	
	Impact of alerts on performance and well-being	Downey et al [32]	

## Discussion

### Principal Findings

RPM is presented as a useful tool to help patients feel safer and more empowered in their self-care during the perioperative period. In addition, health care institutions benefit from it by increasing the efficiency in the use of their resources, both physical (such as beds and monitors) and human (clinical staff). In deciding on the adoption of RPM interventions, considering the impact on and perceptions of clinical staff is crucial as the success of these interventions is based on their cooperation and comprehension. As users and providers of remote perioperative care, clinical staff need to be comfortable and willing to adopt RPM interventions, which should not hinder their other tasks.

Overall, the main RPM-related problems found for clinical staff were related to undesirable changes in their workflow and lack of planning. In several included papers (11/21, 52%), the introduction of RPM led to a higher workload because of unforeseen tasks that emerged when the RPM intervention was implemented in the complex health care environment and not necessarily when the intervention was tested in controlled settings. In particular, tasks such as (remotely monitored) patient data analysis, remote alert response, and remote care reporting and billing were mentioned as sources of increased staff workload and disruptions in the usual care workflow. In addition, the time necessary for activities was often underestimated because of the lack of experience and knowledge of the clinical staff to perform some of the new tasks that RPM interventions created. Furthermore, problems were reported in relation to uncontemplated users as sometimes it was unclear who was in charge of these new tasks, the assigned actor was not the adequate one, or they depended on the assistance of a third party. Problems regarding the difficulty in use and functioning of RPM tools were also described. This was mainly due to lack of knowledge or training, technical malfunctions, or legal issues where the new services conflicted with the current systems. Although it is true that these problems might be temporary and limited to the initial introduction of RPM interventions, it is still important to assess and address them as they do have an impact on the workflow and might cause the intervention implementation to fail before familiarization and adaptation are even possible. Furthermore, it is important to consider initial workflow problems as adaptation strategies and coping mechanisms adopted by staff to overcome these problems might in themselves generate structural issues. For example, when new tasks are introduced by RPM interventions without a clear indication of who is responsible for them, the available actors will feel compelled to take over, adding to their daily workload.

Most of the reported benefits for clinical staff related to the improvement in monitoring and data analysis, resulting in better resource management and clinical outcomes. Even though most staff members agree on the advantages these interventions bring in terms of better follow-up of patients and resource allocation, they are still concerned about the extra workload they face.

Regarding best practices and risk-reduction strategies, most of the included studies (18/21, 86%) mentioned the need to strengthen the implementation process of RPM interventions through better planning and improved stakeholder involvement. This way, clinical staff can provide a better overview of their pre-existing work routines and needs so that the new interventions can be better integrated and adapted to their usual workflow rather than the other way around. Other strategies involved establishing protocols to guide the interventions’ use and operations and providing the necessary training to avoid uncertainty and prepare staff. Finally, several included studies (10/21, 48%) stressed the importance of interoperability and ensuring compatibility between the new RPM interventions and the existing tools and processes used by the staff to prevent double work or the emergence of conflicts in the recorded patient data.

Moreover, it is recommended that RPM-related interfaces be user-friendly and tested in the context to reduce time spent on training and possible technical problems. Enhancing staff’s understanding of and familiarity with the tools can increase their willingness toward their adoption as technology will be perceived as an enhancer and not as an obstacle.

The included studies reported recommendations for best practices and risk-reduction strategies for most of the staff-related problems and challenges mentioned in connection with RPM interventions. It is important to note that these solutions address problems that represent major barriers to RPM implementation in the present. Therefore, adopting them more consistently in RPM research and practice represents a way to maximize the capability of RPM to deliver real-world results in health care services in the future.

Figure 2 shows the connections between themes and categories. Here, we can see how some of the identified themes were not present in all the categories. Notably, there are problems that lack specific recommendations in the literature, such as those related to health care resources. Reimbursement schemes prioritizing in-hospital care constitute a largely unaddressed challenge complicated by the complexity of the context and the different types of stakeholders involved. This affects the commitment and motivation of clinical staff toward RPM interventions as it is not always clear how the extra or new work will be reimbursed. In addition, there are currently few answers on how to increase funding for RPM interventions (Table 1). This is a big challenge, as RPM interventions may not clearly present benefits justifying their relatively high expenses, especially in the short term.

There is still room for improvement in ways to manage incoming alerts so that they do not create interruptions and annoyance among staff while ensuring timely responses. Another open challenge is related to providing a collaborative environment between the different staff members involved in patient care and defining clear roles so as to divide RPM-related tasks effectively and avoid confusion. In addition, there are opportunities to improve the devices and systems that collect, analyze, and communicate patient data. This includes the possibility of using data for more informed or automatized decisions that consider multiple data sources, thus avoiding biases, false positives, and incorrect inputs.

Most of the methods used to assess the impact of RPM interventions on staff-related workflows were qualitative and subjective, including interviews, questionnaires, and observations. Few reported studies (7/21, 33%) included the collection of quantitative measures such as tracking the time invested in using the interventions. This is characterized as an opportunity for improvement in RPM-related research as quantitative impact measures would help assess resource use and, therefore, better evaluate the overall interventions. Furthermore, quantitative measures could unlock the possibility of meaningful comparisons across different interventions and contexts. Some of these more quantitative or objective measures could be anxiety levels using existing scales, as proposed by Jukic et al [42].

However, there is still not enough research on methods to track RPM-related workload quantitatively. Examples of RPM interventions in fields other than perioperative care can be useful in this regard. For example, in tele–intensive care units and the remote monitoring of cardiovascular implantable electronic devices, diverse methods have been deployed to measure staff workload [43-46] by, for example, time-motion studies [47,48]. In these interventions, systems automatically record use time while an observer also tracks the nurses and annotates the duration of RPM-related activities. This has reportedly helped researchers identify the most time-consuming aspects of RPM-related workflow and find bottlenecks and weaknesses to improve designs and implementation plans. These tele–intensive care unit and cardiovascular implantable electronic device remote monitoring research methods could be profitably translated to perioperative care. In general, research on RPM interventions [31,35] helps in understanding possible outcomes and identifying barriers, facilitators, and recommendations [30], which can guide the design and implementation stages of these interventions.

Further research should be dedicated to the quantification of resource use in RPM interventions—to standardize reimbursement policies—and the evaluation of the implementation of these strategies in different settings. Moreover, the time horizon of these studies should be extended to cover longer periods, as many relevant effects of RPM interventions cannot be observed in the short term - partly because of factors such as the staff learning curve.

### Limitations

This scoping review has several limitations. The first is the diversity of RPM interventions examined as they might have different objectives, leading to variable results and problems. In addition, the results will be influenced by the initial state and environment in which the RPM intervention was introduced. As mentioned by Herdman [49], intervention benefits depend on the baseline, whereby an initial higher performance may lead to a comparatively smaller advantage. Moreover, these interventions were executed under different circumstances and environments, which might change the dynamics among the clinical staff. Additional limitations are derived from the differences in the methodology used in the included studies as the target variables and outcomes might not be comparable. Finally, most of the included studies (13/21, 62%) only considered short- and midterm impacts, whereas RPM interventions can have long-term effects that are decisive to assess their overall performance.

This review was also susceptible to risk of bias because of missing results. This risk is increased by our exclusive focus on articles in English, our use of 2 databases (PubMed and Embase), and our focus on a limited time frame (January 2015 to March 2021). Nonreporting bias risk is also likely to apply to this review as we noticed that only a small fraction of papers in the RPM domain reported any insight at all on the impact of the introduction of new interventions on staff workflow. Overall, in light of the aforementioned limitations and risks of bias, we recommend interpreting and using our contribution as an initial description of the types of workflow-related implications of RPM described in the current literature and not as an exhaustive overview.

### Conclusions

Every day there are more studies that show the impact of RPM interventions given their increasing use in clinical practice and in perioperative care in particular. Most of these studies focus on the patient’s perspective and on clinical outcomes. In our scoping review, we presented an overview of the recent knowledge regarding clinical staff’s perspective, which reveals the possible problems and benefits that remote monitoring can bring. Further research regarding policy making and protocol standardization should be conducted to establish a more trustworthy analysis of RPM interventions.

Studies concerning the impact of RPM strategies on clinical staff workflows and dynamics should be clear about the study objective, the design, and the methods used to test the intervention. This will help future readers in assessing the overall performance of RPM interventions. Moreover, this can enable better comparative research and promote the establishment of valuable benchmarking and auditing systems.

## Appendix

Multimedia Appendix 1 Search strategy.

## Abbreviations

PRISMA-ScR: Preferred Reporting Items for Systematic Reviews and Meta-Analyses extension for Scoping Reviews
RPM: remote patient monitoring
