# Supplementary material for: The Impact of Perioperative Remote Patient Monitoring on Clinical Staff Workflows: Scoping Review
Source: JMIR Hum Factors. 2022 Jun 6;9(2):e37204. doi: 10.2196/37204 (PMC9210199; doi:10.2196/37204)
Supplement: Multimedia Appendix 1 [file humanfactors_v9i2e37204_app1.docx]

**Multimedia Appendix 1: Search strategy**

The keywords defined for the search strategy should be found in the summary or title or be one of the main subjects of the document. Also, some global terms, commonly used for certain healthcare topics, were used (“MeSH” and “Emtree”).

**Pubmed**

1. Concept 1: Remote Patient Monitoring (RPM)

"Telemedicine"[majr] OR "Telemonitoring" [Tiab] OR "Remote Monitor*" [Tiab] OR "Telehealth" [Tiab] OR "Remote follow-up" [Tiab] OR "eHealth" [Tiab] OR "Remote Consultation" [Tiab] OR "Remote Sensing Technology" [Mesh] OR "Self-monitor*" [Tiab]

1. Concept 2: Workflow

"Workflow" [Majr] OR "Outcome and Process Assessment, Health Care" [Majr] OR "Task Performance and Analysis" [Majr] OR "Workflow" [Tiab] OR "staffing" [Tiab] OR "Attitude of Health Personnel" [Tiab] OR "alarm fatigue*" [Tiab] OR "alert fatigue" [Tiab] OR "professional burnout" [Tiab] OR "workload" [Tiab] OR "Patient Care Management" [Mesh] OR "Nursing Process*" [Mesh] OR "Clinical Competence" [Mesh] OR "Caregiver Burden" [Mesh] OR "Time and Motion Studies" [Tiab] OR "Work Simplification" [Mesh] OR "Practice Patterns, Nurses'" [MeSH] OR "Nursing Audit"[Mesh]

1. Concept 3: Perioperative care

"Surgical Procedures, Operative" [Majr] OR "General surgery" [Majr] OR "Perioperative" [Tiab] OR "Surgery" [Tiab] OR "Post-operative" [Tiab] OR "post-discharge" [Tiab]

**Embase**

1. Concept 1: Remote Patient Monitoring (RPM)

exp *telemedicine/ or exp *remote sensing/ or (telemonitoring* or Remote Sensing Technology* or Remote Monitor* or Telehealth* or Remote follow-up* or eHealth* or Remote Consultation* or Self-monitor*).ti,ab,kw.

1. Concept 2: workflow

exp *workflow/ or exp *outcome assessment/ or *exp task performance/ or exp *caregiver burden/ or exp *nursing audit/ or exp *patient care/ or exp *clinical competence/ or exp *health personnel attitude/ or (workflow* or outcome assessment* or task performance* or caregiver burden* or nursing audit* or patient care* or clinical competence* or health personnel attitude* or staff* or professional burnout* or workload* or nursing process* or clinical competence* or time and motion studies* or work simplification* or practice patterns*).ti,ab,kw.

1. Concept 3: Perioperative care

exp *surgery/ or exp *general surgery/ or exp *perioperative medicine/ or (surgery* or general surgery* or perioperative* or post-operative* or post-discharge*) .ti,ab,kw.
